# Comment on “The Effect of Cochlear Size on Cochlear Implantation Outcomes”

**DOI:** 10.1155/2020/5674547

**Published:** 2020-07-29

**Authors:** M. Bilgin Eser, Başak Atalay, M. Tayyar Kalcıoğlu

**Affiliations:** ^1^Department of Radiology, Istanbul Medeniyet University Faculty of Medicine, 34722 Istanbul, Turkey; ^2^Department of Otorhinolaryngology & Head and Neck Surgery, Istanbul Medeniyet University, Faculty of Medicine, 34722 Istanbul, Turkey

With great enthusiasm, we read the paper by Kuthubutheen et al. [1] titled “The Effect of Cochlear Size on Cochlear Implantation Outcomes” published recently in BioMed Research International. This study evaluated the cochlea “A” value and outer wall cochlear duct length (CDL) at 720 degrees. The study was aimed at determining if these metrics could be reliably factored into determining hearing outcomes from two different length electrodes. Due to increased cochlear implant surgery, this topic has become a hotspot in otology and radiology. One strong side to the paper is that there is no previous literature discussing different implant outcomes in the cochlea of equal length. Another strength of the study was the evaluation of patients with clinical speech outcomes.

Earlier literature shows that the choice of patient-specific cochlear implants is associated with hearing outcome [2–4]. However, the authors implanted different electrodes into two groups with equal CDL length [1]. Also, unlike the literature, the authors found a relationship between cochlear length and implant success in the group in which they applied short electrodes [5]. It is unlikely that this result can be explained with electrode selection because there are results in the literature that suggest that smaller cochleae may contain fewer spiral ganglion cells [6, 7]. If the short electrode was selected for the long cochlea, the implant success would be expected to be unsatisfactory due to the patient's incompatibility with specific tonotopy [5]. Although not statistically significant, the CNC score shift between the two groups is in favor of Flex 31, and this is not emphasized in the discussion [1].

In Materials and Methods, the authors mention that they measure the first 720-degree outer wall of the cochlea. Although the whole outer wall length of the cochlea is challenging to measure accurately, it is not impossible [8]. The measurement of the apical turn also contributed to the literature because this is the least studied part of the cochlea [8, 9]. Thus, the actual measurement could be shared instead of the estimated full-length value in the discussion. The authors claim that the absence of validation of histopathology by imaging, which they specify as a limitation of the technique, is not relevant. However, Adunka et al., Würfel et al., and Timm et al. tested the reliability of the method with histopathology and imaging [4, 6, 10].

In the presentation of Table 3, a material error was noted. As could be seen in Figure 2, the CDL length was measured as 32.289 mm at 720 degrees. However, the combined value for 360 degrees is 32.29 mm, and for 720 degrees is 21.3 mm. Another topic relates to the presentation of statistical results. In Figure 3, *p* values were not shared for Pearson correlation coefficients. Although these *p* values are given roughly in Table 4, sharing the exact values in Figure 3 would increase reliability [1].
